# β-Amyloid Plaque Reduction in the Hippocampus After Focused Ultrasound-Induced Blood–Brain Barrier Opening in Alzheimer’s Disease

**DOI:** 10.3389/fnhum.2020.593672

**Published:** 2020-10-07

**Authors:** Pierre-François D’Haese, Manish Ranjan, Alexander Song, Marc W. Haut, Jeffrey Carpenter, Gerard Dieb, Umer Najib, Peng Wang, Rashi I. Mehta, J. Levi Chazen, Sally Hodder, Daniel Claassen, Michael Kaplitt, Ali R. Rezai

**Affiliations:** ^1^Rockefeller Neuroscience Institute, West Virginia University, Morgantown, WV, United States; ^2^Department of Electrical Engineering and Computer Science, Vanderbilt University, Nashville, TN, United States; ^3^Department of Neurological Surgery, Vanderbilt University, Nashville, TN, United States; ^4^Department of Neuroradiology, West Virginia University, Nashville, TN, United States; ^5^Department of Neurosurgery, West Virginia University, Nashville, TN, United States; ^6^Department of Neurology, Vanderbilt University, Nashville, TN, United States; ^7^Department of Behavioral Medicine and Psychiatry, West Virginia University, Morgantown, WV, United States; ^8^Department of Neurology, West Virginia University, Morgantown, WV, United States; ^9^Department of Radiology, Weill Cornell Medical College, New York, NY, United States; ^10^West Virginia Clinical and Translational Science Institute, West Virginia University, Morgantown, WV, United States; ^11^Department of Neurological Surgery, Weill Cornell Medical College, New York, NY, United States

**Keywords:** Alzheimer’s disease, focused ultrasound, FUS, β-amyloid reduction, hippocampus, blood-brain barrier

## Abstract

The blood–brain barrier (BBB) limits therapeutic delivery in Alzheimer’s disease (AD) and other neurological disorders. Animal models have demonstrated safe BBB opening and reduction in β-amyloid plaque with focused ultrasound (FUS). We recently demonstrated the feasibility, safety, and reversibility of FUS-induced BBB opening in the hippocampus and entorhinal cortex in six participants with early AD. We now report the effect of BBB opening with FUS treatment on β-amyloid plaque. Six participants underwent ^18^F-Florbetaben PET scan at baseline and 1 week after the completion of the third FUS treatment (60 days interval). PET analysis comparing the hippocampus and entorhinal cortex in the treated and untreated hemispheres revealed a decrease in the ratio of ^18^F-Florbetaben ligand binding. The standard uptake value ratios (SUVr) reduction ranged from 2.7% to 10% with an average of 5.05% (±2.76) suggesting a decrease in β-amyloid plaque.

## Introduction

Alzheimer’s disease (AD) is the most common form of dementia with progressive memory and cognitive decline. The absence of any effective treatment for AD has led to experimental studies of immunotherapy targeting the amyloid-beta plaque and other novel therapeutics (Cummings et al., 2019). A significant limitation in these treatments is the presence of the blood–brain barrier (BBB). Previously, it was reported that low-intensity MR-guided focused ultrasound (FUS) can open the BBB in the frontal lobe in patients with Alzheimer’s disease (Lipsman et al., 2018). Recently, we demonstrated that FUS can safely and reversibly open a large portion (average 29%) of the hippocampus and entorhinal cortex (EC) BBB in patients with early AD (Rezai et al., 2020). Animal studies have shown that FUS BBB opening reduced β-amyloid plaque and improved memory (Burgess et al., 2014). The impact of BBB opening on β-amyloid plaque remains unclear in humans. In this study, we report the effect of FUS treatment in the hippocampus and EC on β-amyloid plaque in six participants.

## Materials and Methods

### Study Design

**FIGURE 1:**
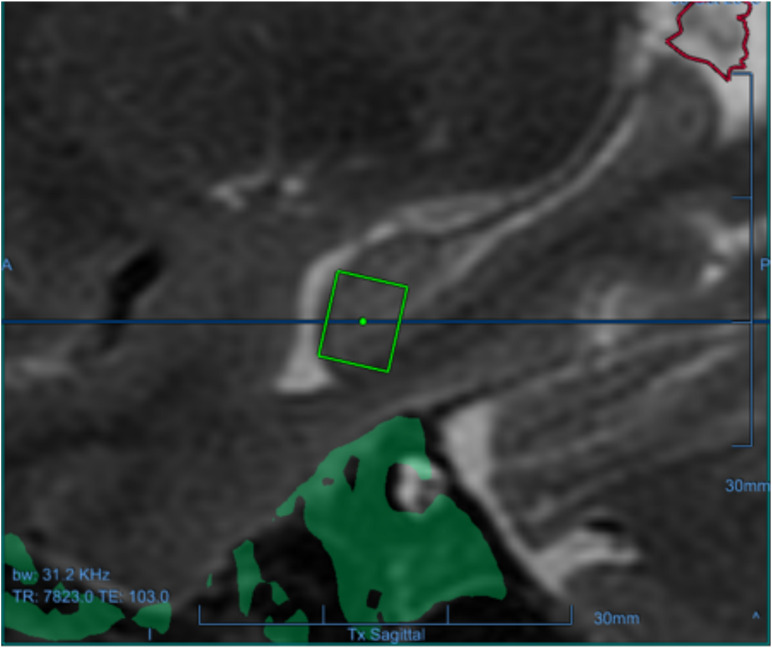
One of the targets for one of the participants as a green square in the left hippocampus, overlaid on the baseline sagittal T1-weighted MRI.

**FIGURE 2:**
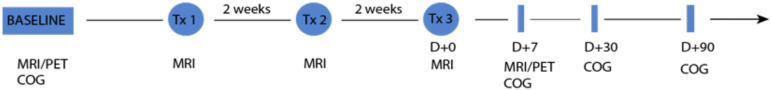
Timeline of the experiments. The pair of PET and MRI images is acquired at baseline and 7 days after the third and last sonication treatment. Cognitive state of the patient is assessed at baseline, 7, 30, and 90 days after the last sonication. Each patient undergoes three sonication treatments 2 weeks apart.

This is an open-label, prospective phase II clinical trial (NCT03671889) (Website) explicitly designed to target the memory structures of the hippocampus and EC in humans with early AD. The overall objective of the study was to evaluate the safety, feasibility, efficacy, and effect on β-amyloid plaque from repeated BBB opening of the hippocampus and EC in AD. The trial was sponsored by INSIGHTEC (Haifa, Israel). The protocol was approved by the institutional review boards. Eligible participants were between 50 and 85 years of age with early AD diagnosed by a multidisciplinary team according to the National Institute of Aging Alzheimer’s Association (NIA-AA) criteria (McKhann et al., 2011) and had evidence of β-amyloid on ^18^F-Florbetaben positron emission tomography (PET) scans (Rowe et al., 2008). Participants underwent FUS treatment at either the West Virginia University Rockefeller Neuroscience Institute or Weill Cornell Medical College with 220 kHz ExAblate Neuro Type 2 system (INSIGHTEC) to the hippocampus/EC with concomitant IV microbubble (Definity) injection with a cumulative max dose of 20 μl/kg immediately prior to sonication using a range of power of 4–11.5 W for 90 s. The sonication power and parameters were tailored to each subject to achieve a 50–60% of cavitation thresholds to open the BBB. FUS targets were selected either in the left or right hippocampus/EC, based on individual anatomy and β-amyloid plaque distribution (Rezai et al., 2020) (Figure 1). In this study, we use ^18^F-Florbetaben as the PET β-amyloid imaging agent (Rowe et al., 2008) to facilitate the clinical evaluation and provide an objective measure of the FUS treatment on the β-amyloid plaque burden. Each participant received three FUS-induced BBB opening sessions 2 weeks apart and underwent ^18^F-Florbetaben PET-CT imaging at baseline and 1 week after the third session (approximately 60 days from baseline) (Figure 2). PET analysis was performed after alignment of PET/CT and MRI using an Automatic Mutual Information-based Registration (Lavely et al., 2004). Pre-treatment MRI was used to segment the hippocampi and EC using a non-linear atlas-based algorithm (Rohde et al., 2003; Lavely et al., 2004; Chakravorti et al., 2019). The standard uptake value (SUV) is a mathematically derived ratio of tissue radioactivity concentration at a point in time and the injected dose of radioactivity per kilogram of the participant’s body weight and is used to assess the activity in PET imaging. The ratio of the SUV from two different regions, a target and reference region, within the same PET image is commonly abbreviated as SUVr. While other references have been studied (Klein et al., 2015), we follow a commonly accepted approach to assess longitudinal changes of β-amyloid plaque burden in a subject by using the SUVr obtained with the cerebellar gray matter as the reference tissue and comparing it with SUVr at baseline (Ossenkoppele et al., 2012). In addition, since the treatment was unilateral, we evaluated the ratios between the SUVr of the treated (SUVr(T)) and the untreated (SUVr(NT)) hippocampus/EC of each participant. Given that SUVr(T) (SUV(T)/SUV(Cerebellum)) and SUVr(NT) (SUV(NT)/SUV(Cerebellum)), the ratios are computed as ratio (SUV(T)/SUV(CR))/(SUV(NT)/SUV(CR)) (SUV(T)/SUV(NT)), which is equivalent to computing the SUVr with the untreated side as the reference instead of the cerebellum. To assess the impact of FUS treatment on β-amyloid plaque upon the target region of a focal unilateral intervention, we evaluated the treated/untreated SUVr ratios, assessing differences among the two sides at baseline and after treatment.

## Results

**FIGURE 3:**
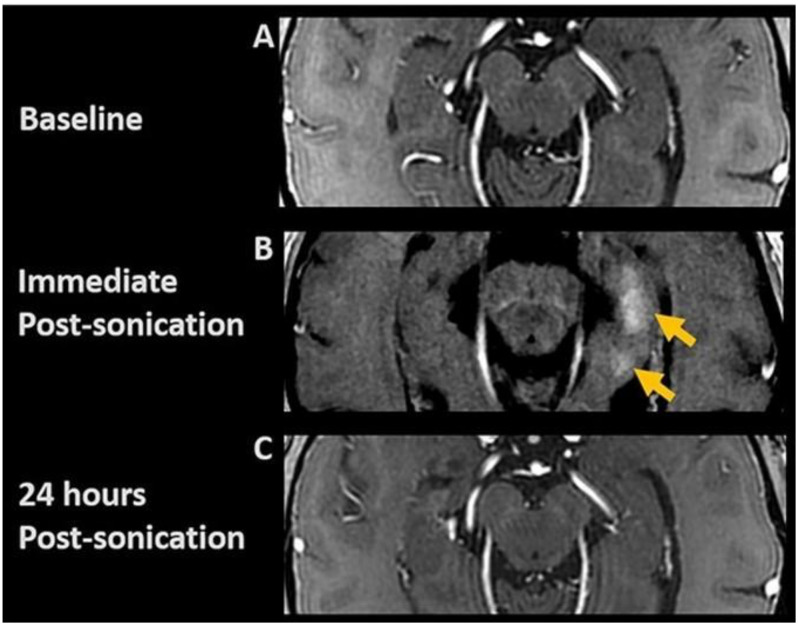
Post-contrast axial T1-weighted images. **(A)** Baseline; **(B)** Immediate post-FUS treatment demonstrating contrast enhancement (arrows) at target sites, indicating focal BBB opening; **(C)** 24-h post-sonication images show resolution of parenchymal contrast enhancement, indicating BBB closure.

Six participants (55–73 years of age, five females, one male) completed the trial. For reference, in all 18 treatment sessions (three per participant), parenchymal contrast enhancement was demonstrated at each target with an average opening of 95% of the FUS target volume corresponding to 29% of the overall hippocampus volume and resolved in 24 h indicating opening and closure of BBB (Figure 3) (Rezai et al., 2020). Conventional cerebellar-based PET analysis on the treated hippocampus/EC shows after treatment a change from baseline of −12.44%, −5.78%, −1.83%, −1.70%, 0.11%, and −17.15%, respectively, for participants 1 to 6. For reference, the change from baseline on the untreated hippocampus/EC is −9.36%, 0.49%. 0.94%, 3.17%, 3.34%, and −7.88% (Table 1). The PET analysis using the contra-lateral side as a reference revealed decreases in the SUV ratio of treated/untreated in all participants. Post-treatment changes in the SUVr for participants one through six were −3.4%, −6.2%, −2.7%, −3.3%, −4.7%, and −10%, respectively (Table 2), suggesting a decrease of the β-amyloid plaque with an average reduction of 5.05% (±2.76). Formal cognitive assessments did not reveal any clinically meaningful changes at the time of the PET imaging.

**TABLE 1:** Impact of the FUS treatment through conventional analysis using the cerebellum as the reference region to compute the SUVr.

	SUVr-treated hippocampus/EC			SUVr untreated hippocampus/EC		
Patient ID	Baseline^a^	Post-treatment^b^	% change from baseline^c^	Baseline^d^	Post-treatment^e^	% change from baseline^f^
1	1.792	1.569	−12.44%	1.72	1.559	−9.36%
2	1.471	1.386	−5.78%	1.438	1.445	0.49%
3	1.799	1.766	−1.83%	1.705	1.721	0.94%
4	1	0.983	−1.70%	1.011	1.043	3.17%
5	0.905	0.906	0.11%	1.048	1.083	3.34%
6	1.265	1.048	−17.15%	1.294	1.192	−7.88%

*Columns indicates, respectively, the SUVr for the treated and the untreated hippocampus/EC for each participants at baseline (a and d) and post-treatment (b and e). The percentage change from baseline (c and f).*

**TABLE 2:** Impact of the FUS treatment through SUVr asymmetry analysis.

	Treated/untreated hippocampus/EC asymmetry		
Patient ID	Baseline^a^	Post-treatment^b^	% change^c^
1	1.041	1.007	−3.35%
2	1.023	0.959	−6.23%
3	1.055	1.026	−2.72%
4	0.989	0.943	−4.69%
5	0.864	0.836	−3.25%
6	0.977	0.879	−10.05%

*Asymmetry is computed as the ratio of SUVr (cerebellum as the reference region) for the treated and the untreated hippocampus/EC for each participant. Asymmetry is presented at baseline (a) and post-treatment (b). Changes of asymmetry(c) are presented as percentage change between the baseline and post-treatment ratios of uptake value.*

## Discussion

There is a crucial need for new treatment strategies for Alzheimer’s disease as basic research and clinical trials exploring effective and novel treatment options have failed despite extensive efforts. The BBB is an inherent challenge for systemic delivery of medication, immunotherapy, genetic treatment, and other therapeutics to the brain. FUS disruption of the BBB alone in animal models has resulted in a reduction in β-amyloid plaque burden (Burgess et al., 2014). The improvements and clearance of the plaque by FUS may result from structural opening of the BBB facilitating clearance, modulation of the immune system, or potentially enhanced lymphatic clearance. This report presents the first human results of the effects of opening the BBB on the reduction of β-amyloid plaque in the hippocampus and EC. In our PET analysis, the focal and unilateral nature of the treatment provided the opportunity to standardize the PET uptake value ratios using the untreated contralateral hippocampus as the control. Reduced β-amyloid deposition was evident post-procedure in all six participants on the treated side. A common approach for PET analysis is to standardize uptake value ratios (SUVr) using the cerebellar cortex as a reference region since this is often not subject to the same processes that affect supratentorial gray matter, particularly when global interventions may influence much of the brain. Our approach for PET analysis is a validated approach and commonly used in mesial temporal lobe epilepsy seizure analysis (Neuroimaging Subcommision of the International League Against Epilepsy, 2000). While it is possible that a change in the untreated hemisphere could confound this data, it is unlikely that any substantial natural progression of the disease would occur over the 60-day study period. In addition, any natural variation in ligand binding would be unlikely to occur in the same direction in all six participants treated on different sides and at two different institutions. It is possible that the same result could be obtained with stabilization of β-amyloid binding on the FUS treated side and an increase of β-amyloid binding on the untreated side. While unlikely, this would also be of potential therapeutic value but would represent a mechanism of stabilization rather than improvement.

In summary, these preliminary results are encouraging and suggest that FUS BBB opening is not only feasible and safe but may induce reduction in β-amyloid plaque burden. These findings are consistent with the animal studies. If reproducible and sustainable in humans along with stabilization or improvement of the disease, FUS may hold promise for a new therapeutic option for AD in the future.

## Data Availability Statement

The raw data supporting the conclusions of this article will be made available by the authors, without undue reservation.

## Ethics Statement

The studies involving human participants were reviewed and approved respectively by the West Virginia University’s and Weill Cornell Medical College’s IRB. The patients/participants provided their written informed consent to participate in this study.

## Author Contributions

AR, MK, and P-FD’H conceived the presented idea. AR, MK, JLC, JC, MR, RM, UN, and MH contributed to the medical approach of the treatment. P-FD’H, AS, and DC developed the theory and performed the computations and interpretation of the results. AR, MK, and SH supervised the findings of this work. AR, MK, and SH verified the analytical methods. All authors discussed the results and contributed to the final manuscript.

## Conflict of Interest

The authors declare that this study received funding from INSIGHTEC, INC. The funder was not involved in the study design, collection, analysis, interpretation of data, the writing of this article, or the decision to submit it for publication.
